# An Adaptive and Spectrally Efficient Multi-Channel Medium Access Control Protocol for Dynamic Ad Hoc Networks

**DOI:** 10.3390/s22228666

**Published:** 2022-11-10

**Authors:** Abdurrahman Beg, Saud Mohammad Mostafa, AbdulAziz AbdulGhaffar, Tarek R. Sheltami, Ashraf Mahmoud

**Affiliations:** 1 Faculty of Information Technology, Monash University, Melbourne 3800, Australia; 2 Department of Computer Engineering, King Fahd University of Petroleum and Minerals, Dhahran 31261, Saudi Arabia; 3 Department of Systems and Computer Engineering, Carleton University, Ottawa, ON K1S 5B6, Canada; 4 Interdisciplinary Research Center of Smart Mobility and Logistics, King Fahd University of Petroleum and Minerals, Dhahran 31261, Saudi Arabia

**Keywords:** medium access control, multi-channel MAC protocol, ad hoc networks, adaptive MMAC, wireless communication

## Abstract

Medium access control (MAC) protocols in ad hoc networks have evolved from single-channel independent transmission mechanisms to multi-channel concurrent mechanisms to efficiently manage the demands placed on modern networks. The primary aim of this study is to compare the performance of popular multi-channel MAC (MMAC) protocols under saturated network traffic conditions and propose improvements to the protocols under these conditions. A novel, dynamically adaptive MMAC protocol was devised to take advantage of the performance capabilities of the evaluated protocols in changing wireless ad hoc network conditions. A simulation of the familiar MAC protocols was developed based on a validated simulation of the IEEE 802.11 standard. Further, the behaviors and performances of these protocols are compared against the proposed MMAC protocols with a varying number of ad hoc stations and concurrent wireless channels in terms of throughput, Jain’s fairness index, and channel access delay. The results show that the proposed MMAC protocol, labeled E-SA-MMAC, outperforms the existing protocols in throughput by up to 11.9% under a constrained number of channels and in channel access delays by up to 18.3%. It can be asserted from these observations that the proposed approach provides performance benefits against its peers under saturated traffic conditions and other factors, such as the number of available wireless channels, and is suitable for dynamic ad hoc network deployments.

## 1. Introduction

In recent times, there has been an exponential growth of network-capable devices wirelessly connecting to the grid. Devices communicate over the internet through various means, such as 5G cellular [1,2], Wi-Fi, ZigBee, and other network technologies. The advent of technologies, such as the Internet-of-Things (IoT) [3], gives physical objects of any size IP addresses, and has sprouted further explorations into novel applications, such as connected cars and the internet of drones [4,5]. These wireless applications have primarily two methods of connecting to the network, infrastructure-based or peer-to-peer ad hoc-based, and in some cases, a hybrid of both technologies.

Wireless local area networks (WLANs) enable devices to communicate without requiring physical cable links connecting the network. The building blocks of WLANs involve the basic service set (BSS). The BSS can have two different architectures based on the presence or absence of the access point (AP) [6,7].

- *Ad hoc network*: Does not contain any APs and devices communicate without it.
- *Infrastructure network*: The AP is the centralized node and all traffic flow through it.

The focus of this study is on ad hoc network characteristic-decentralized topologies with nodes communicating as peers between themselves. In an ad hoc network, a single channel needs to be shared by several nodes, and the medium access control (MAC) protocol [8,9,10] is responsible for the coordination between nodes. With advances in technological development, the physical (PHY) layer is vastly improved with higher data rates and a better quality of service (QoS) [11,12,13,14]. Although IEEE 802.11 allows the use of multiple channels, IEEE 802.11 MAC protocol is used for handling channel access in a single channel only. Moreover, it is found to be inefficient due to multi-channel hidden terminal problems, which will be discussed later. The approaches to MMAC protocols have been classified as follows [15,16,17]:

- Dedicated control channel;
- Split phase;
- Common hopping;
- Parallel rendezvous.

The dedicated control channel approach splits the spectrum into one control channel and N-1 data channels where each node has two radio interfaces to operate in the control and data channel. Nodes are aware of the neighbor transmissions and no synchronization is needed. Due to the common control channel, no multi-channel hidden problem exists.

The split phase, as the name suggests, divides the time into two phases—the contention and the data transfer phase. These two phases are fixed and always take place periodically. During the contention phase, all nodes listen to the common control channel, and those that succeed exchange data frames in the data exchange phase. Although the multi-channel hidden terminal is solved and tight global synchronization is needed.

In common hopping, nodes follow a hopping sequence. The nodes that want to communicate stop at a frequency and communicate and then rendezvous with the hopping sequence. Hopping spreads the control signal and congestion is reduced on a particular channel, but tight global synchronization is also needed in this approach.

A parallel rendezvous is similar to the common hopping approach but multiple handshakes occur simultaneously on all free channels.

Although the parallelism of MMAC protocols significantly improves throughput and reduce channel access delay compared to a single-channel approach [15], there are issues that need to be addressed. The issues that reside in MMAC protocols are as follows:

- *The multi-channel hidden terminal* occurs when two nodes are busy in the data transmission in one channel and are unaware of control packets sent by other nodes that want to communicate on a particular channel. When these nodes return to the control channel, they might want to communicate on that busy channel, causing a collision [18,19].
- *The missing receiver* occurs when the control packets sent to a receiver are not acknowledged due to it being busy in the data transmission with another node [20].
- *Deafness* occurs when the intended receiver is busy in the data transmission and ignores the control packets sent to it [21].

Generally, the radio antennas of the wireless nodes can operate in one of two modes, omnidirectional or directional. Nodes using omnidirectional antennas can listen to communications and transmit data in all directions. Using omnidirectional antennas is beneficial since the nodes do not need to direct their antennas in a specific direction for communication. This makes omnidirectional antennas easy to install and less expensive. However, the disadvantage of these antennas is that there is less signal strength, which reduces their range. The chances of collision are also higher in this case because the nodes listen to communications from all directions. On the other hand, nodes using directional antennas can only communicate in a specific direction. Using this antenna provides higher connectivity and a longer range. Moreover, it reduces interference and allows multiple communications in close vicinity without interference. Nevertheless, with directional antennas, nodes have a deafness problem. The overall cost and complexities of these antennas are higher than omnidirectional antennas [22,23,24].

The objective of this research is to improve the performance and efficiency of the MMAC protocol and compare it with other protocols with varying factors, namely, the number of stations and channels. The primary contributions of this work are as follows:

- We propose an enhanced spectrally efficient asynchronous multi-channel MAC (E-SA-MMAC) protocol that utilizes the spectrum efficiently and increases the overall throughput in constrained environments.
- The proposed protocol solves the issues of the multi-channel hidden terminal and deafness by using omnidirectionally broadcast control packets.
- Further, a second protocol is proposed called Max-E-SA-MMAC, by modifying the backoff algorithm of the E-SA-MMAC, to improve fairness between the nodes.
- An adaptive MMAC protocol is proposed; we took advantage of the performance characteristics of the evaluated protocols by opting for the more ideal transmission mechanism in the changing network environments.
- An extensive performance evaluation of the proposed algorithms was carried out under three performance metrics—normalized throughput, Jain’s fairness index (JFI), and channel access delay.
- A full factorial design with analysis of variation (ANOVA) was also performed to analyze the impacts of the factors on the results.

The rest of the paper is organized as follows. Section 2 comprises the literature review of different multi-channel MAC protocols that were studied for this research. The proposed solution of this paper is presented in Section 3 followed by the simulation, results, and performance evaluation discussion in Section 4, Section 5 and Section 6, respectively. Lastly, the conclusion and future work will be presented in Section 7.

## 2. Literature Review

Numerous research was conducted on multi-channel MAC protocols that focus on the use of multiple channels to enhance network performance [25,26,27]. Few researchers have proposed an energy-efficient MMAC protocol [28,29,30,31], whereas other proposed schemes ensure reliability and fairness [32,33,34]. In this section, we highlight the protocols that were used in this study. The basic contention mechanism of the IEEE 802.11 standard for WLANs has been used in many MMAC protocols. Therefore, we begin with the discussion of the contention mechanism in IEEE 802.11 that uses Carrier-sense multiple access with collision avoidance (CSMA/CA) [35,36,37]. The nodes always sense the medium before transmitting to avoid a collision. Each node selects a random backoff time that is based on uniform distribution between [0, CW) where CW denotes the current contention window. When the channel is found idle, the node decrements the backoff time, and upon reaching 0, transmits its frame. If it finds the channel busy, it pauses its backoff timer until it finds the medium idle again. Upon collision, CW is doubled until $CW_{max}$ and attempts to transmit the frame until the retry limit is reached. Upon successful transmission, CW is set to $CW_{min}$ and nodes wait for the DIFS (distributed coordination function interframe space) [38] time to sense the channel again. The receiver also acknowledges the (ACK frame) reception of data after waiting for the SIFS (short interframe space) time. The issue in this protocol is the time spent in backoff and contention that reduces the throughput of the network [39,40].

MMAC improves the throughput significantly by having nodes transmitting on multiple channels [41]. All channels of the spectrum are orthogonal to each other, meaning transmission in one channel would not interfere with the transmission in another channel. In MMAC protocols, the contention is basically in the control channel and any two nodes winning the contention and deciding on the channel to use, switch to that channel for data transmission. The nodes contend using control frames in the control channel [16].

The first MMAC protocol considered in this work is a bidirectional multi-channel MAC (BiMMAC) [42,43], which uses the IEEE 802.11 MAC protocol to contend in the control channel. Multiple channels are used in BiMMAC where one channel is exclusively used for the control channel and others are used for the data channel. The process of data transmission is divided into two phases, namely the control phase and the data exchange phase. In the control phase, control frames, such as request-to-send (RTS) and clear-to-send (CTS) [44], are exchanged to negotiate the channel that can be used for data transmission. Another control frame named channel reservation (CRN) is used by the source to announce data transmission to all neighbors of the source to avoid the multi-channel hidden problem. In the data exchange phase, both nodes switch to the negotiated channel for data transmission. After the data are successfully transmitted, the nodes switch back to the control channel. The main feature of BiMMAC is the transmission of two frames in the data channel with a single handshake, i.e., if both nodes have packets to send to one another then they will exchange data frames without the need to contend again. In case the receiver has no data to send, it can send a normal ACK and both nodes switch to the control channel. Due to the extra frame sent by the receiver without contention or control frame exchanges, control overhead is reduced and network throughput is increased. The drawback of this protocol is that the nodes suffer large delays due to the waiting time in exchanging two frames.

The authors of [45] proposed a collision-free asynchronous MMAC for low-cost and more power-efficient nodes that have a single half-duplex transceiver. One common control channel is used and the rest are used as data channels. The asynchronous multi-channel MAC (AMMAC) differs from the above two protocols due to the reuse of the control channel for data transmission. Contending nodes in the control channel send RTS-CTS control frames, such as other protocols, and also send an additional announce-to-send (ATS) frame to inform the neighbors of both the sender and receiver of the intended data transmission. Nodes switch to the data channel that has been agreed upon during the contention in the control channel. When there is no data channel available, the nodes that win the contention are allowed to send one frame in the control channel. Spectrum utilization is increased due to the use of the control channel for data transmission. Due to this behavior, one mandatory waiting frame time is added to nodes coming back to the control channel for contention. Although an overhead, this ensures nodes returning to the control channel do not collide with ongoing transmission. Therefore, the issue of the multi-channel hidden terminal is solved due to this asynchronous mode of operation. However, the disadvantage of this scheme is that nodes returning to the control channel need to wait a mandatory period before transmitting frames.

The authors of [46] propose a hybrid and adaptive H-MMAC protocol, which improves the AMMAC protocol in utilizing the control channel for data transmissions as well as extending data transmissions in data channels. Further, H-MMAC utilizes an ad hoc traffic indication message (ATIM) of the IEEE 802.11 power saving mode (PSM) for power management. Using the ATIM window, nodes negotiate data transmission sessions and exchange data/ACK packets while the other nodes go into sleep mode. H-MMAC defines data transmissions in two modes: normal and extended modes. Normal transmissions occur within the data window and extended transmissions extend to the ATIM window based on the network traffic load. The extended mode can continue to the next data window if other nodes do not use the specific data channel. For control channel reuse, the control channel cycles between two intervals (contention and data intervals). In contention intervals, nodes negotiate with RTS-CTS packets, and in data intervals, nodes that successfully negotiate data transmission slots exchange data and ACK packets.

The spectrally efficient asynchronous multi-channel MAC (SA-MMAC) [47] enhances the spectrum utilization by reusing the control channel for data transmission, such as AMMAC. If no free data channels are found, the node can transmit one frame in the control channel. Moreover, it follows the bidirectional data transmission mechanism explained in BiMMAC. SA-MMAC uses one common channel and the rest as data channels out of all orthogonal channels. The contention mechanism in the control channel is similar to IEEE 802.11 MAC protocol. The control frames used during contention are RTS-CTS-ATS and solve the issue of multi-channel hidden terminal problems. Once the nodes negotiate which data channel to use, they switch to it and start the data transmission. The receiver can send its frame without contention, backoff, or RTS-CTS handshakes. After data transmission, the nodes return to the control channel where they wait for a mandatory one frame of time to avoid collision with ongoing transmission in the control channel. SA-MMAC reduces the signaling overhead and increases the utilization of the spectrum. Moreover, it solves the deafness and multi-channel hidden terminal problem. Delay incurred by the other nodes in obtaining the channel would be high due to two frames in the data channel. Moreover, nodes need to wait an additional frame of time before they contend due to data transmission in the control channel.

## 3. Proposed Solution

The proposed solution is divided into two sections: the first section describes the proposed protocol for high network load situations and the second presents a novel dynamic approach to utilize the strengths of the MMAC protocols simulated in this research for various wireless ad hoc network situations.

### 3.1. E-SA-MMAC and Max-E-SA-MMAC

The proposed solution, an enhanced SA-MMAC (E-SA-MMAC), extends the SA-MMAC protocol. E-SA-MMAC uses one common control channel and the rest as data channels out of all the orthogonal channels. The contention takes place in the control channel and successful nodes switch to negotiated data channels for data transmission.

The control frames exchanged in the control channel are as follows:

- *RTS*: This frame is sent when a node has data to send and it also has additional information about the free channels.
- *CTS*: This frame is sent by the receiver to inform the sender of its reception of the RTS frame and it includes the channel that has been selected for data transmission.
- *ATS*: This frame is used to announce to the neighbors of both the sender and receiver nodes about the data transmission and the negotiated data channel that will be used by these nodes.

**Figure 1:**
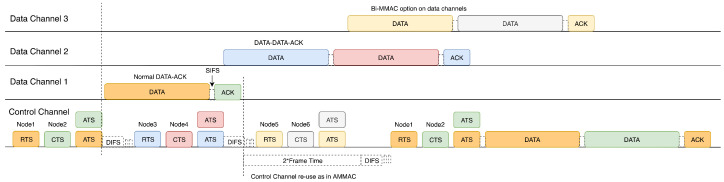
Timing diagram for proposed MMAC protocol.

The proposed protocol adopts the idea of bidirectional data exchange between nodes in the data channel as well as the control channel to maximize the throughput of the network during peak traffic conditions. Figure 1 shows the timing diagram for the proposed protocol with four channels (one control and three data channels). The operation of the protocol is as follows:

- Nodes contend in the control channel and successful nodes send RTS.
- Upon reception of RTS, the receiver replies with a CTS specifying the selected channel to use for data transmission. Moreover, it starts a timer, and upon expiration of this timer, it sends ATS.
- Upon reception of CTS, the sender checks the selected channel and sends ATS.
- Both nodes switch to the negotiated data channel after sending ATS and they commence data transmission. Two frames can be exchanged bi-directionally if the receiver also has data to send.
- If there are no available data channels, the nodes transmit in the control channel. In this protocol, we allow the transmission of two frames in the control channel.
- After data transmission, nodes return to the control channel and wait for two mandatory frame times to avoid collision with any ongoing transmission in the control channel.

**Figure 2:**
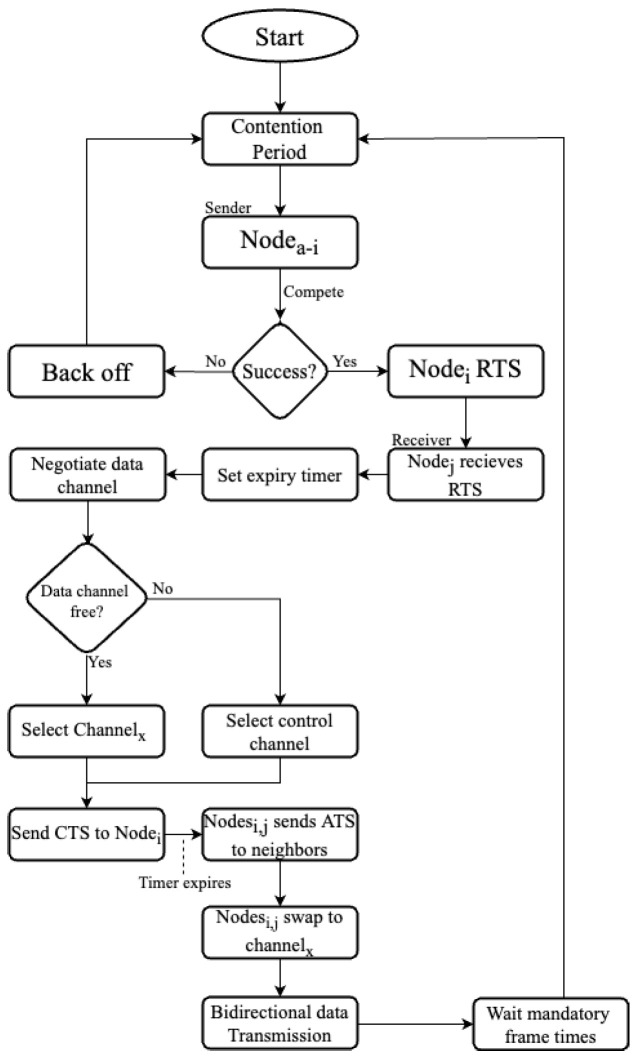
Flow diagram of the proposed E-SA-MMAC protocol.

The above protocol operation is described in detail in Figure 2. After the initial contention period, the node that succeeds sends the RTS to the receiver, which then negotiates a data transmission channel, if not in operation. If all the data channels are busy, then the protocol allows this pair of nodes to transmit data in the control channel, bi-directionally, unique to this protocol.

This protocol utilizes spectrum more efficiently and throughput increases for high contention cases. The deafness and multi-channel hidden terminal problems are solved using the RTS-CTS-ATS exchange and mandatory waiting time. However, the delay incurred for nodes would be higher as more waiting time is needed for nodes to access channels. Additionally, compared to SA-MMAC the fairness would be lower due to two frames being sent in the control channel, increasing the waiting time for queued nodes. In addition to E-SA-MMAC, we propose Max-E-SA-MMAC, which has the same features as the originally proposed protocol but the backoff algorithm has been modified. Initially, the nodes start with [0, $CW_{min}$), and upon success, remain uniformly distributed between this range. In case of collision, the contention window is set to $CW_{max}$ and nodes choose random backoff times based on uniform distribution [0, $CW_{max}$). This mechanism ensures fairness between nodes with higher throughput from SA-MMAC. Table 1 shows the comparison of different protocol features.

### 3.2. Adaptive MMAC

In this section, the authors present a novel approach to utilize the performance capabilities of the simulated MMAC protocols in different situations. In Section 5.2, the analysis shows that E-SA-MMAC and its variant Max-E-SA-MMAC perform the best in terms of throughput and fairness in three or fewer channels due to the utilization of the control channel for data transmission. However, when presented in environments with a greater number of wireless channels, say twelve, the control channel becomes the bottleneck as more data transmission channels are available for negotiation. If the negotiation is interrupted due to the data transmission on the control channel, data transmission dedicated channels are under-utilized as the “handshake” process is delayed. Therefore, the authors propose a dynamic MMAC, which we refer to as adaptive MMAC, which adapts to the number of wireless channels available and uses the control channel for data transmission, only when there are three or fewer channels. Otherwise, it performs the BiMMAC routine of using the control channel for contention mechanisms only.

**Figure 3:**
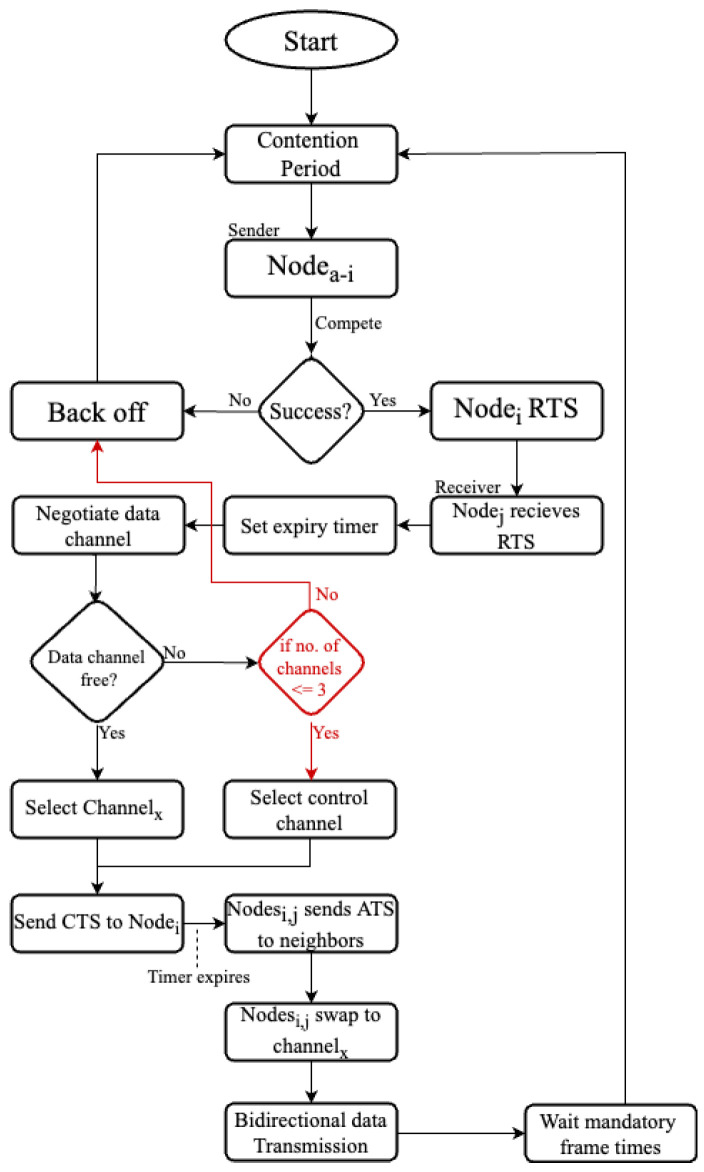
Flow diagram of the proposed adaptive MMAC protocol.

Figure 3 describes the flow diagram of the Adaptive MMAC protocol. The portion in red is the difference between this protocol and the E-SA-MMAC protocol, enabling the protocol to behave differently based on the resources available.

## 4. Simulation

### 4.1. Simulation Environment and Design

The simulation was developed following the conceptual model described by the flow diagrams in Figure 2 and Figure 3 in the proposed solution. The initial step was to design a modular code with subsystems interacting with each other to mimic the baseline IEEE 802.11 protocol and to verify the accuracy of the coding method. The simulation environment is done on Python 3 with each protocol compartmentalized in separate python files. A parent python program calls each protocol to execute simultaneously with a given set of configurations, such as the maximum number of stations, increment increase in stations per round, number of wireless channels available, contention window, and other parameters described in the simulation parameters.

Each protocol python file involves running functions that mimic a network of a varying number of stations competing to access a wireless channel to transfer data to another station. This is modeled using array structures consisting of frames up to the maximum number of frames allowed as per the predefined simulation time. Another array is configured with random backoff periods from the 0 to $CW_{max}$ window for each collision that occurs. Collisions are modeled as situations when multiple stations select the same frame time to send the packet, and a collision counter is assigned to each station in another array to store historical contention results. This collision counter imposes a greater backoff penalty for stations to ensure fairness in the algorithm. Nodes fairing badly with subsequent collisions are not able to access a wireless data transmission channel, thus access delay increases and fairness decreases.

After the contention mechanism, the model moves to the success event function with an assigned data transmission channel for two frame time units. This function, depending on the protocol, determines whether the station receiving the transmission is able to send a bidirectional data transmission in the same transmission window post-handshake. This function is the primary difference in the code between the protocols, with the exception of E-SA-MMAC and Max-E-SA-MMAC, as they differ in contention windows during a failure event scenario.

### 4.2. Simulation Parameters

Table 2 describes the parameters for the simulation of this research. For simulator validation, this work considers comparing the saturated throughput trend of IEEE 802.11 MAC protocol in the experiment with the original work presented in [48]. The payload, headers, and control packet sizes were kept the same as in the previous literature to maintain consistency in measurement. The number of stations ranged from 5 to 70, with increments of 5 stations, until saturation was reached. Further, the simulation was configured with IEEE 802.11b, which allows three concurrent channels without overlap by having three usable channels in the simulation. Similarly, IEEE 802.11n had 4 non-overlapping channels, and an upper-bound case for 12 usable channels was also simulated [49,50]. In addition, the results are verified by comparing the normalized throughput of SA-MMAC to confirm the correctness of the Python-based simulation.

### 4.3. Host Specifications

Table 3 and Table 4 describe the physical host and software specifications, respectively. The simulation environment was developed on Python 3 and executed on the Ubuntu Linux operating system, hosted in a VMware virtual machine.

The following assumptions are made for the simulation scope of the experimental environment and, subsequently, the performance evaluations of the results:

- The nodes are in a single collision domain and are in the transmission range of each other.
- The traffic pattern is in a high-load situation, where all nodes have data packets to send and contend for reservation of a channel.
- The data rate is considered to be at a fixed rate of 1 Mbps for both data and control packets.
- Simulations run for 10,000 data frames.

**Figure 4:**
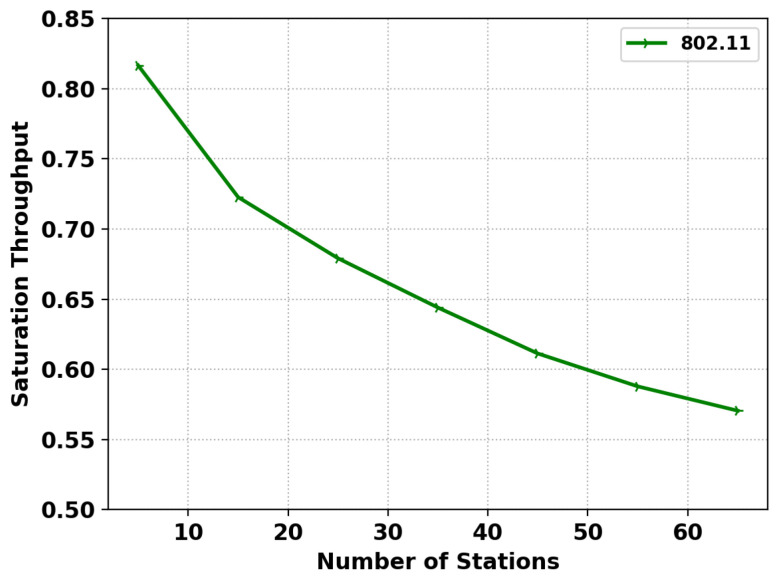
Simulation of IEEE 802.11.

**Figure 5:**
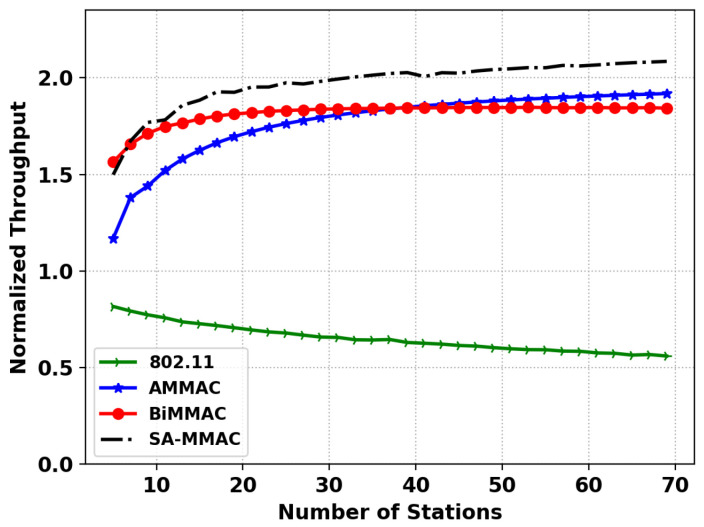
Simulation of IEEE 802.11, AMMAC, BiMMAC, and SA-MMAC.

**Figure 6:**
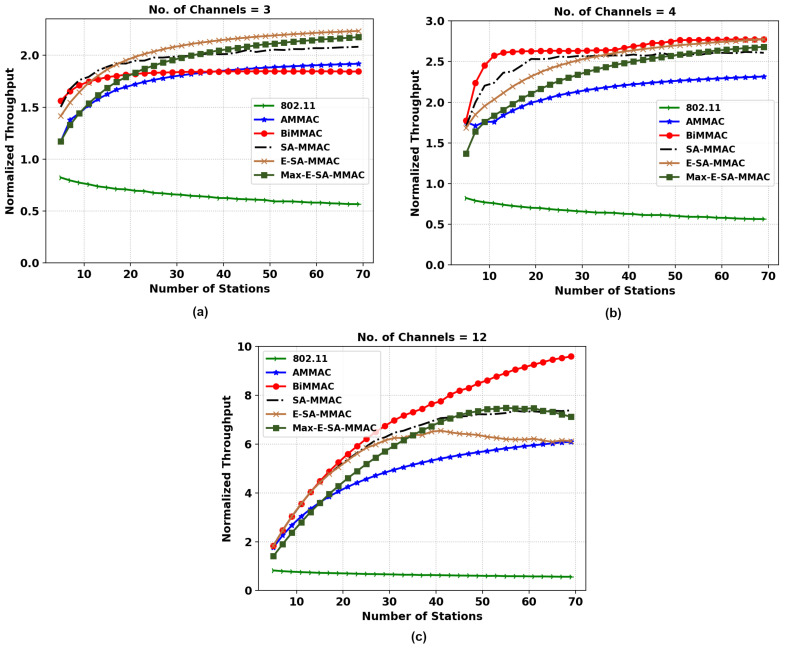
Normalized throughput comparison of the two MMAC protocols, E-SA-MMAC and Max-E-SA-MMAC, versus others; (**a**) 3, (**b**) 4, and (**c**) 12 channels.

Figure 4 shows the saturated throughput of the IEEE 802.11 MAC protocol in the simulator, which has a similar trend to the results of (Figure 6) in [48] and in this simulation. Figure 5 shows the normalized throughput for SA-MMAC, AMMAC, BiMMAC, and IEEE 802.11 in the developed simulator to compare against (Figure 4) in [47]. As both the figures match the trend in results of the original works, we are confident this simulation produces the correct results with high precision.

## 5. Results

### 5.1. Performance Metrics

The following performance metrics are measured in this simulation:

- *Normalized throughput*: This is calculated by dividing the total number of packets received ($P_{total}$) by the number of packets that can be sent in a given simulation time ($T_{sim}/F$), and then multiplying it by the ratio of the packet payload ($L_{pkt}$) to the frame size (*F*). Where $T_{sim}$ represents the maximum simulation time and *F* indicates the frame size. Normalized throughput is calculated using Equation (1).
  (1) $$Normalized\;Throughput=\left[\frac{P_{total}}{\left(\frac{T_{sim}}{F}\right)}\right]\times \left[\frac{L_{pkt}}{F}\right]$$
- *Jain’s fairness index (JFI)*: Average fairness among nodes queued for channel access. This is obtained using Equation (2), where *n* is the number of stations and *t* is the time for the current experiment.
  (2) $$\begin{array}{c} Average\;Fairness\;Index=\left[\frac{\sum_{t}\left(\frac{{\left(\sum_{i=1}^{n}x_{i}\right)}^{2}}{n.\left(\sum_{i=1}^{n}x_{i}^{2}\right)}\right)}{t}\right] \end{array}$$
- *Channel access delay*: Average queuing delay for a node to access the channel. The channel access delay is computed from Equation (3), where *N* is the number of stations and $d_{n}$ is the queuing delay of packets of nth station.
  (3) $$\begin{array}{c} Channel\;access\;delay=\frac{\sum_{n=1}^{N}d_{n}}{P_{total}\times 1000} \end{array}$$

### 5.2. Analysis

#### 5.2.1. Normalized Throughput

Figure 6 of the throughput analysis shows the performance of the multi-channel MAC (MMAC) protocols including the IEEE 802.11 as a base reference to compare with the single-channel protocol. It is clear that multi-channel MAC protocols (in the case of high-load scenarios) have significantly better throughput results than IEEE 802.11. The concurrency of data packets delivered by multi-channel MAC protocols explains the large margin of difference in performance.

The figures compare the MMAC protocols in configurations of 3, 4, and 12 channels with the number of stations varying by steps of 2 nodes from 5 to 70 nodes. In Figure 6a, it can be seen that bidirectional MMAC (BiMMAC) outperforms the other protocols initially with a low number of stations in a three-channel network. However, once the number of stations increases, the performance decreases in BiMMAC as it saturates early while other protocols continue to improve the network throughput. This can be explained by the lack of control channel utilization for data transmissions by the BiMMAC protocol.

Both of the proposed protocols, E-SA-MMAC and Max-E-SA-MMAC, outperformed all other protocols in this scenario. This can be explained by the fact that in high-load situations, where every station has a packet to send all of the time, maximizing each RTS-CTS handshake by using the bidirectional packet transfer has a significant impact on the number of successfully transmitted data packets. In the proposed protocols, this includes the use of a control channel to send data packets in both directions when other dedicated data channels are currently unavailable.

In Figure 6b, the throughput pattern changes significantly. BiMMAC has a better throughput initially; however, with a larger number of stations, the proposed protocol is able to match BiMMAC as it saturates due to the lack of control channel utilization for data transmission. The multi-channel MAC protocols using data transfer on the control channel perform poorly due to the extra overhead and longer backoff timers in the control channel. The fairness index of the former protocol, BiMMAC, falls sharply after approximately 40 stations while E-SA-MMAC and Max-E-SA-MMAC remain more fair and consistent, which will be explained under JFI analysis.

In Figure 6c, the multi-channel MAC protocols of 12 channels suffer similarly to 4 channels due to the extra overhead and longer backoff timers in the control channel as the station numbers increase. Therefore, protocols not using data transfers in the control channel are able to negotiate more promptly the RTS-CTS demands and keep their data channels occupied. After a certain point, however, it is expected that the number of stations contending in a dedicated control channel will eventually collide more often than successfully negotiating a data channel; thereby, we expect a decrease in the overall network throughput. This is a disadvantage of a dedicated control channel.

#### 5.2.2. Fairness

**Figure 7:**
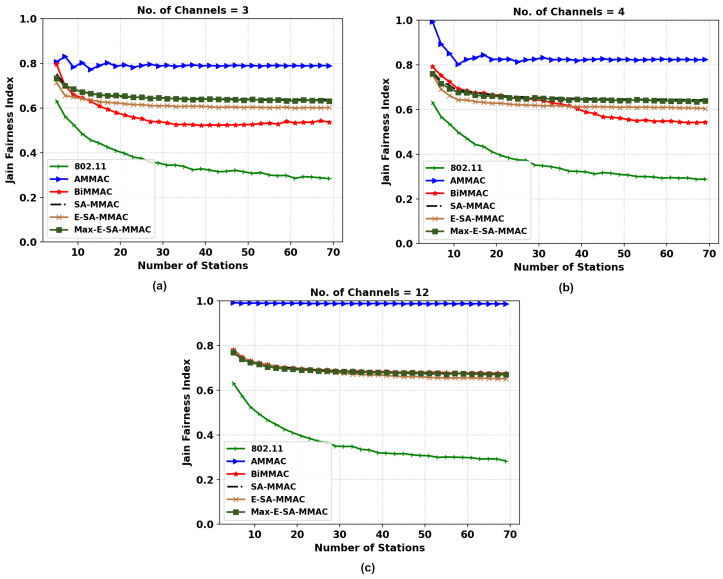
JFI comparison of the two MMAC protocols, E-SA-MMAC, and Max-E-SA-MMAC, versus others; (**a**) 3, (**b**) 4, and (**c**) 12 channels.

Figure 7a shows the results of Jain’s fairness index (JFI) versus the number of stations for a three-channel configuration. The proposed protocol, whilst providing a higher throughput with the same number of available channels, proves to have a lesser Jain’s fairness index than the original SA-MMAC. Therefore, we proposed an enhancement to the protocol, Max-E-SA-MMAC, which has a higher throughput than SA-MMAC whilst providing a similar JFI performance. Max-E-SA-MMAC assigns $CW_{max}$ for nodes that have successfully transmitted data frames, allowing for those in the queue to reach the end of their backoff timer faster and gain access to the channel, improving fairness.

In the 4-channel configuration, Figure 7b, we notice that the BiMMAC loses significant fairness performance after the number of stations passes 35. However, both the proposed protocols and SA-MMAC have a consistent fairness index even as the number of stations increases and remains fairer than the BiMMAC protocol. The queued nodes wait a longer period of time to access the channel in BiMMAC compared to our protocols due to the lack of control channel utilization for data transmission.

In Figure 7c, all MMAC protocols apart from AMMAC remain consistent and relatively equal. The AMMAC protocol in all three configurations has the highest JFI due to the nature of the protocol only sending one data packet per handshake, allowing for more nodes to send single packets. This however comes at the cost of a poorer throughput performance in a saturated network with a high load of data packets to be sent. For other protocols, except IEEE 802.11, the JFI remains equal due to the availability of a greater number of data channels; therefore, there are fewer stations queued for data transmission.

#### 5.2.3. Channel Access Delay

**Figure 8:**
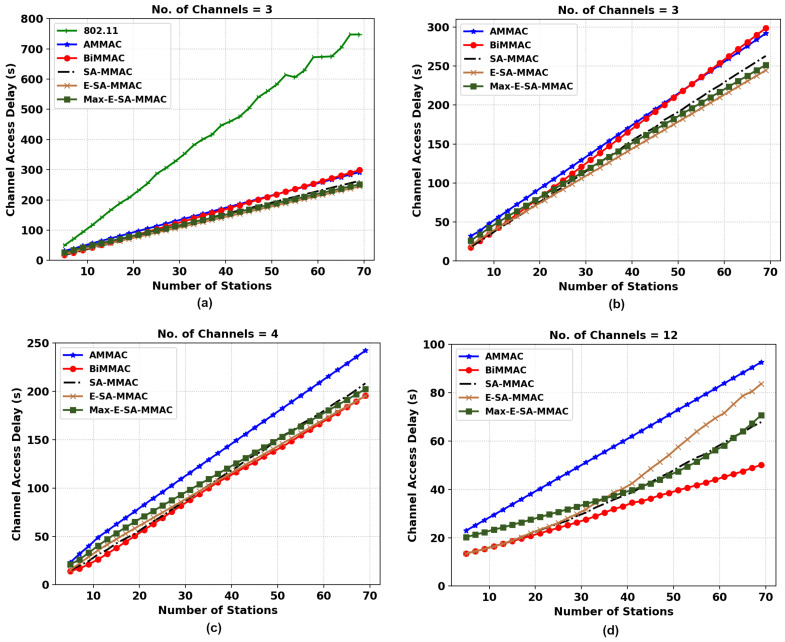
Channel access delay comparison of the two MMAC protocols, E-SA-MMAC, and Max-E-SA-MMAC, versus others. (**a**) Three channels with IEEE 802.11, (**b**) three channels, (**c**) four channels, and (**d**) twelve channels.

Figure 8a describes the channel access delay between the MAC protocols for a three-channel configuration. It was measured that the channel access delay for IEEE 802.11 was approximately 206% higher than the E-SA-MMAC protocol at 70 stations. This is due to IEEE 802.11 having only a single channel for data transmission, incurring a greater channel access delay for stations that have packets to send. To better represent the differences among the MMAC protocols, we removed IEEE 802.11 from the analysis.

In Figure 8b, the E-SA-MMAC protocol has the lowest channel access delay, followed by the second protocol, Max-E-SA-MMAC, then SA-MMAC, and the other protocols. This can be attributed to the pure bidirectional mode of operation, even in the control channel, with the protocol. More stations have a chance to piggyback a data packet with the acknowledgment.

In Figure 8c, AMMAC has the highest delay in channel access, whilst the other protocols are fairly equal in performance. The reason for this, as explained previously, is due to the single data packet transmission per handshake in the AMMAC protocol, while the other protocols allow for bidirectional data transmission per handshake. Figure 8d shows BiMMAC with the lowest channel access delay as the number of stations increases due to less congestion at the control channel.

### 5.3. Analysis of Adaptive MMAC

#### 5.3.1. Normalized Throughput

**Figure 9:**
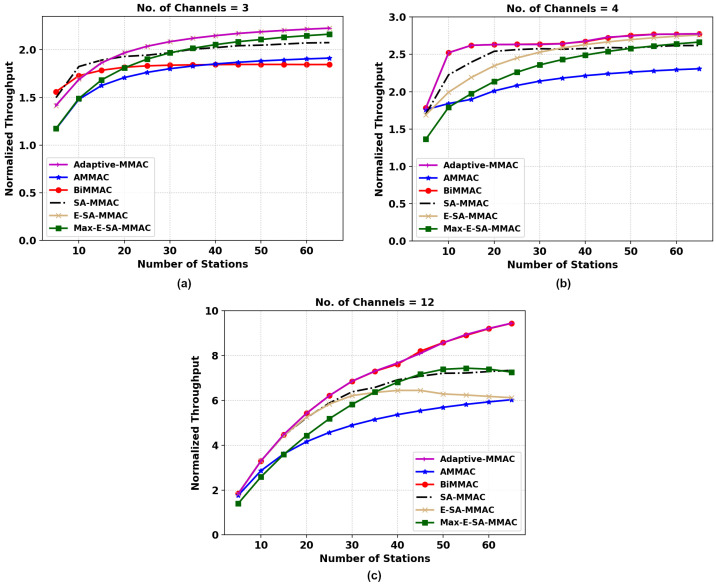
Normalized throughput comparison of adaptive MMAC versus others. (**a**) Three, (**b**) four, and (**c**) twelve channels.

Figure 9 describes the results of the adaptive MMAC protocol that dynamically changes to behave similarly to the E-SA-MMAC and BiMMAC protocols depending on the wireless ad hoc channels availability. Figure 9a shows the adaptive MMAC protocol switching to the E-SA-MMAC mode when there are only three available data channels. The protocol provides the best throughput figures as it enables the control channel to be used for data transmission when all the other channels are busy. Figure 9b,c shows the adaptive MMAC switching to the BiMMAC mode and disabling data transmission in the control channel as that would block negotiations for the two mandatory frame time backoff. By opting for the BiMMAC mode, only control packets are transmitted in the control channel, negotiating available data transmission channels.

#### 5.3.2. Fairness

**Figure 10:**
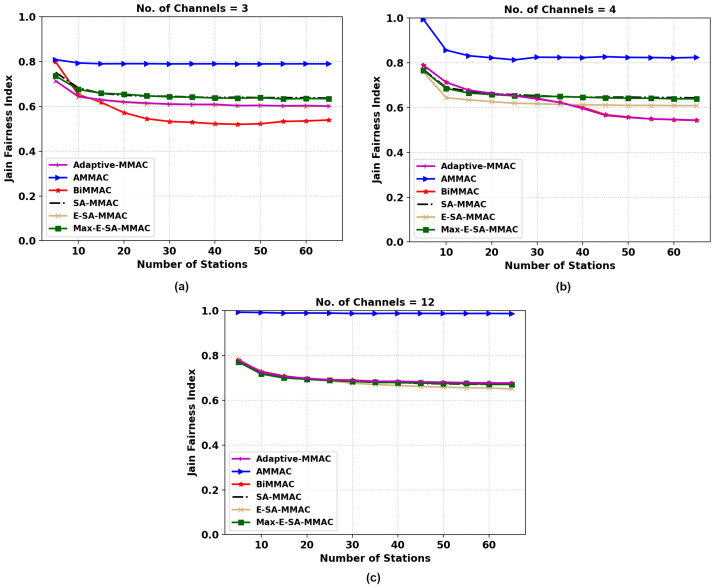
JFI comparison of adaptive MMAC versus others. (**a**) Three, (**b**) four, and (**c**) twelve channels.

Figure 10 describes the JFI metric of the adaptive MMAC protocol. Similar to the previous analysis of AMMAC, it is the fairest of the protocols evaluated in this study. However, in this instance, the adaptive MMAC of the three channel conditions seen in Figure 10a is similarly as fair as E-SA-MMAC, as it takes that mode. It can be seen in Figure 10b,c that when adaptive MMAC takes the BiMMAC route, it is similarly fair to that protocol. Overall, the trend is that the new protocols converge to a similar fairness level of 0.7 JFI as the number of channels and stations increase.

## 6. Performance Evaluation

For the performance evaluation, we used a full factorial design with k = 3 factors [52,53]. The factors we selected were the MMAC protocols, number of stations, and number of channels. Each factor contains different levels to consider. Table 5 shows the factors and the levels considered for the MMAC performance study.

$y_{ijkl}$ = response (observation) in the Ith replication of the experiment with factors A = MMAC protocols, B = No. of channels, and C = No. of stations at levels i = 5, j = 3, and k = 4, respectively.

μ = mean response

$\alpha_{i}$ = effect of factor A at level i

$\beta_{j}$ = effect of factor B at level j

$\xi_{k}$ = effect of factor C at level k

$\gamma_{AB_{ij}}$ = interaction between A and B at levels i and j.

$\gamma_{ABC_{ijk}}$ = interaction between A, B, C at levels i, j, k.

From the analysis of variation (ANOVA), Table 6, of the throughput analysis, we notice the main effect on the throughput variation is mostly due to the number of channels with almost 56% variation and, to a lesser extent, the number of stations with an impact of around 20.6% of the results, with the MMAC protocols having the least impact with a variation of 1.6%. These variations were calculated from the respective ANOVA tables by dividing the sum of squares of each factor by the total.

Additionally, the interaction between the number of channels and the number of stations has the most significant effect with a 17.7% variation in the throughput compared to any other interaction combination. This implies that the number of channels and the number of stations match each other in terms of the effect they have on the throughput. The interaction between all three factors accounts for only 1.4% of the variation.

Table 7 describes the ANOVA for JFI, which highlights the impact of the MMAC protocols on the variation of the fairness index. The MMAC protocols contribute 58.7% to the variation in the results. The number of stations has the next most impactful effect with 20.3%, but the number of channels has a negligible effect on fairness.

Moreover, the table shows that the interaction between MMAC protocols and the number of channels has the largest effect with a 4.7% variation in the fairness from the interacting factors. Interactions between all factors account for 2.4% of the variation.

Table 8 describes the ANOVA for channel access delay, which shows that the impact of the number of stations in the collision domain of the network has the most significant effect on the variation of channel access delay with 55.8%. The second most impactful effect is the number of channels with 28.5%, while the MMAC protocols explain the least percentages of variation.

Furthermore, the interaction between the number of stations and channels explains the greatest percentage of variation among the interactions of the factors with almost 13%. This implies that the number of stations and channels match each other in terms of the effect they have on the channel access delay. The interaction between all three factors accounts for only 0.4% of the variation and, therefore, is negligible.

**Figure 11:**
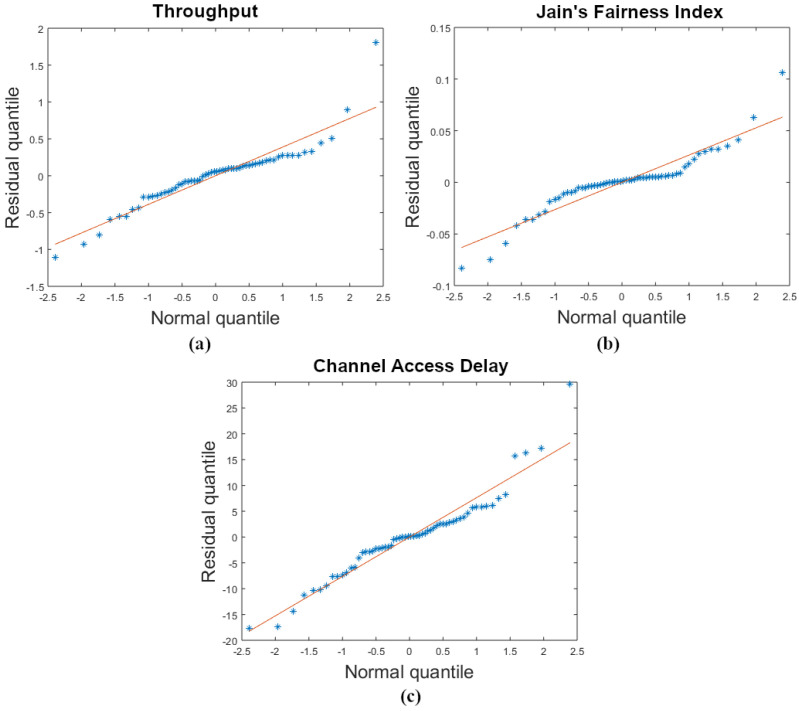
Quantile–quantile plot of (**a**) throughput, (**b**) JFI, and (**c**) channel access delay.

The assumption that the residuals are normally distributed is proven by the quantile–quantile plots for all three factors in Figure 11a–c. The visual tests show that the residuals fall evenly around the least squares line.

## 7. Conclusions

In conclusion, the proposed E-SA-MMAC and Max-E-SA-MMAC protocols achieve greater throughput than the other MMAC protocols at the expense of fairness while the number of channels is low. Max-E-SA-MMAC adapts to the $CW_{max}$ mode, which provides greater throughput and equal fairness to the previous works. A large number of channels shows a negative effect on the throughput of the protocol due to the congestion at the control channel. Thus, we extended the protocol to adapt to the changing wireless ad hoc network conditions based on the number of channels by using the control channel for data transmission at a low availability of channels and the BiMMAC approach in other situations. The results of this adaptive MMAC demonstrate that this approach achieves the best of both worlds in dynamically changing environments.

Further, we learned from the analysis of variation that protocols have a much smaller effect on the variation of throughput compared to the number of stations and the total number of channels. The number of channels has the greatest effect on the variation of throughput by a large margin. The ANOVA table for throughput also highlights the effect of the interaction between the number of channels and the number of stations on the variation of throughput. Lastly, MMAC protocols explain the majority of the variation in fairness, while the number of stations contributes to the majority of the channel access delay.

### Future Work

For future work, the contention window should be adaptable, depending on the network sizes, and considering the mobility of nodes. Moreover, an optimization approach can be used to optimize the value of $CW_{max}$ used in Max-E-SA-MMAC. Security of the protocol can be tackled by the use of the lightweight cryptographic algorithms at the MAC layer. Furthermore, the simulation environment and subsequent parameters should be adjusted to the modern 1 Gbps bit rate of the channel so further tests could be performed to confirm whether the results stay consistent for this simulation scenario. In addition, this study can be extended to consider the size of the collision domain to measure the fairness among nodes in the network.

## Figures and Tables

**Table 1:** Comparison of features.

Feature	IEEE 802.11	BiMMAC	AMMAC	SA-MMAC	E-SA-MMAC
Issue of multi-channel hidden terminal	NA	Very less	No	No	No
Control channel overhead	NA	Less	High	High	Very High
More than one data frame in a handshake	No	Yes	No	Yes	Yes
Number of transceivers	1	1	1	1	1
Exclusive control channel needed	NA	Yes	No	No	No
Channel selection *	Random	Last Used	Random	Last Used	Last Used
* Subject to availability of common channels.					

**Table 2:** Simulation Parameters.

Parameter	Configuration
Packet payload	8224
PHY header	192 bits
MAC header	224 bits
Frame (Payload + PHY + MAC)	8640 bits
RTS	176 bits + PHY header
CTS	128 bits + PHY header
ATS	128 bits + PHY header
ACK	304 bits
Channel Bit Rate	1 Mbit/s
SIFS	10 μs
DIFS	50 μs
$CW_{min}$	32
$CW_{max}$	1024
Number of Stations	5–70
Channels available	3, 4, 12
Maximum simulation time	10,000 frame time

**Table 3:** Physical host specification.

Item	Specification
CPU	Intel Core i5-8250U CPU @ 1.60 GHz
Host Operating System	Windows 10 (64-bit)
Guest Operating System	Ubuntu 14.4 [51]
Virtualization Tool	VMware Workstation 14
Virtual Machines	1
Main Memory	20 GB

**Table 4:** Software specification.

Item	Specification
Platform	Python 3.10.7
Dependencies	NumPy, Matplotlib
Data Analysis	MATLAB R2022a, Microsoft Excel

**Table 5:** Factors and levels for MMAC performance study.

Factors\Levels	1	2	3	4	5
Protocols	AMMAC	BiMMAC	SA-MMAC	E-SA-MMAC	Max-E-SA-MMAC
Number of Channels	3	4	12		
Number of Stations	5	25	45	65	

**Table 6:** ANOVA for normalized throughput.

Component	Sum of Squares	% Variation	Degree of Freedom	Mean Square
*y*	860.0205		60	
$\bar{y}$ …	599.9794		1	
$y-\bar{y}$…	260.0411	100%	59	
**Main Effects**	203.7815	78.36%	9	22.6
No. of Stations	53.6673	20.64%	3	
MMAC Protocols	4.0952	1.57%	4	
No. of Channels	146.019	56.15%	2	
**First Order Interactions**	52.564	20.20%	26	2
Stations*Protocols	1.8193		12	
Stations*Channels	46.0823		6	
Protocols*Channels	4.6624		8	
**Second Order Interactions**	3.6956	1.40%	24	0.2
Stats*Proto*Channels	3.6956		24	

**Table 7:** ANOVA for Jain’s Fairness Index.

Component	Sum of Squares	% Variation	Degree of Freedom	Mean Square
*y*	31.1306		60	
$\bar{y}$…	30.3636		1	
$y-\bar{y}$…	0.767	100%	59	
**Main Effects**	0.6838	89.10%	9	0.1
No. of Stations	0.1556	20.29%	3	
MMAC Protocols	0.4501	58.68%	4	
No. of Channels	0.078	10.17%	2	
**First Order Interactions**	0.0649	8.50%	26	0.0025
Stations*Protocols	0.0192		12	
Stations*Channels	0.0095		6	
Protocols*Channels	0.0362		8	
**Second Order Interactions**	0.0183	2.40%	24	0.00076
Stats*Proto*Channels	0.0183		24	

**Table 8:** ANOVA for Channel Access Delay.

Component	Sum of Squares	% Variation	Degree of Freedom	Mean Square
*y*	904,502.3		60	
$\bar{y}$…	550,405.2		1	
$y-\bar{y}$…	354,097	100%	59	
**Main Effects**	302,778.4	85.50%	9	33,642
No. of Stations	197,759.6	55.85%	3	
MMAC Protocols	4207.245	1.19%	4	
No. of Channels	100,811.6	28.47%	2	
**First Order Interactions**	50,000.7	14.10%	26	1923.1
Stations*Protocols	860.6436		12	
Stations*Channels	47,618.91		6	
Protocols*Channels	1521.139		8	
**Second Order Interactions**	1317.925	0.40%	24	54.9
Stats*Proto*Channels	1317.925		24	
